# Detection of *Borrelia miyamotoi* in *Ixodes nipponensis* in Korea

**DOI:** 10.1371/journal.pone.0220465

**Published:** 2019-07-29

**Authors:** Choon Mee Kim, Ji Won Seo, Dong Min Kim, Na Ra Yun, Jung Wook Park, Jae Keun Chung, Hyun Jae Song

**Affiliations:** 1 Premedical Science, College of Medicine, Chosun University, Gwangju, South Korea; 2 Gwangju Science Academy for the Gifted, Gwangju, South Korea; 3 Department of Internal Medicine, College of Medicine, Chosun University, Gwangju, South Korea; 4 Division of Infectious Disease Investigation, Health and Environment Research Institute of Gwangju City, Gwangju, South Korea; 5 Clinical Pathology, Gwangju Health University, Gwangju, South Korea; Tufts University Cummings School of Veterinary Medicine, UNITED STATES

## Abstract

**Background:**

This study investigated *Borrelia* species prevalence in ticks from vegetation, through a molecular method, in Gwangju Metropolitan City, South Korea.

**Methodology/Principal findings:**

A total of 484 ticks were collected through flagging and dragging in a suburban area of Gwangju Metropolitan City, South Korea, in 2014. These ticks were morphologically identified and subjected to nested PCR, targeting *Borrelia*–specific CTP synthase (*pyrG*), outer surface protein A (*ospA*) and flagellin (*flaB*) genes. Molecular biological species identification of *Borrelia*-positive ticks was conducted via 16S rRNA PCR assays. Of the 484 ticks collected, 417 (86.2%) were identified as *Haemaphysalis longicornis*, 42 (8.7%) as *H*. *flava*, and 25 (5.2%) as *Ixodes nipponensis*. All the ixodid ticks containing *Borrelia* species bacteria were confirmed to be *I*. *nipponensis* adults, by both morphological and molecular methods. Of the 25 *I*. *nipponensis* ticks collected, four (16%) were positive for *Borrelia* species, three of which were *B*. *afzelii* and one *B*. *miyamotoi*.

**Conclusions/Significance:**

Our study has shown the harboring of *B*. *miyamotoi* by *I*. *nipponensis* in South Korea. Morphological and molecular genetic analyses revealed that, in South Korea, *I*. *nipponensis* could potentially transmit *B*. *miyamotoi* to humans.

## Introduction

Vector-borne, infectious diseases can be transmitted via mosquitoes, ticks, fleas, and other vectors, and account for more than 17% of all infectious diseases [1]. Five genera and 27 species of ixodid ticks have been reported in South Korea [2]. Lyme borreliosis is a vector-borne disease, caused by *Ixodes* species having a *Borrelia* infection, occurring mainly in Europe, North America, and Asia. Bacteria of the *Borrelia* -genus, including *Borrelia burgdorferi*, *B*. *afzelii*, and *B*. *garinii*, are known to be causative pathogens of Lyme borreliosis [3, 4]. Of the tick species capable of transmitting Lyme borreliosis pathogens, *I*. *ricinus* is typically found in Europe, *I*. *scapularis* and *I*. *pacificus* are found in North America, and *I*. *persulcatus* and *I*. *nipponensis* in Asia [5–7]. *I*. *scapularis* is the primary vector of *B*. *burgdorferi* (the Lyme disease spirochete), *B*. *miyamotoi* (the *B*. *miyamotoi* disease spirochete), *Anaplasma phagocytophilum* (the causative agents of anaplasmosis), *Babesia microti* (the causative agents of babesiosis), and Powassan virus (the causative agents of Powassan virus infection) [8].

*B*. *garinii*, *B*. *afzelii*, *B*. *valaisiana*, *B*. *japonica*, *B*. *tanukii*, *B*. *turdi*, and *B*. *sinica* have all been detected in Asia [3]. In 2011, the genotypes of 102 *B*. *burgdorferi* sensu lato isolates, from 11 provinces in China, were investigated. Most *B*. *garinii* strains were isolated from *I*. *persulcatus* ticks collected in northern China; however, *B*. *afzelii* strains were found in *I*. *persulcatus* ticks collected in northern China and *H*. *bispinosa* ticks collected in southern China [9]. *B*. *miyamotoi* is also transmitted by *Ixodes* spp. ticks, and unlike the species causing Lyme borreliosis, it has been reported that larvae are able to transmit the pathogen. *B*. *miyamotoi* is transovarially transmitted from adult females to larval offspring, via their eggs [10]. Thus, nymphal, adult ticks, and previously unfed larval *Ixodes* ticks, can transmit the pathogen.

Since its discovery in *I*. *persulcatus* in Japan in 1994 [11], *B*. *miyamotoi* has been detected in various *Ixodes* spp. in North America, Europe, and Russia [4, 12, 13]. In South Korea, *B*. *burgdorferi* sensu lato was detected in *I*. *persulcatus* ticks for the first time in 1993 [14].*B*. *miyamotoi* has, however, never been detected in South Korea and there have been no previous studies of the vector for *B*. *miyamotoi*. In the present study, 484 ticks, collected in a suburban area of Gwangju Metropolitan City, South Korea, in 2014, were morphologically identified and subjected to nested-PCR(N-PCR) targeting the *Borrelia*–specific CTP synthase (*pyrG*) gene and the outer surface protein A (*ospA*) gene. To verify the result using an additional target gene, N-PCR testing using the *Borrelia*-specific flagellin gene (*flaB*) was performed. In addition, mitochondrial 16S rRNA PCR was performed for species identification of *Borrelia*-positive tick species. This is the report of *B*. *miyamotoi* in ixodid ticks in South Korea. Morphological and molecular genetic analyses revealed that, in South Korea, *I*. *nipponensis* could be involved in *B*. *miyamotoi* infections by functioning as a vector.

## Methods

### Collection and identification of ticks

From April to September 2014, five sites within Gwangju Metropolitan City (Mudeung mountain (35°08'07.0"N 126°59'19.1"E), Buk-gu Konkuk-dong (35°13'59.9"N 126°53'11.6"E), Bunjeok mountain (35°06'28.7"N 126°55'12.3"E), Eodeung mountain (35°09’19.2” N, 126°45’05.4” E) and Seo-gu Central Park (35°08'10.9"N 126°51'53.8"E)) in South Korea were selected and ticks were collected by flagging and dragging methods. No specific permissions were required for the collection of ticks; however, permission from landowners was attained as the collections were conducted on private property. The field studies did not involve endangered or protected species. Recovered ticks were kept in containers labeled with the location, date, and collection time. Life cycle stages and species of all collected ticks were morphologically identified using microscopy and standard taxonomic keys [15].

### PCR detection and molecular identification of *Borrelia* species and ticks

The ticks were washed with 70% ethanol for 2–5 min, washed again with sterilized distilled water for 3 min, dried on sterile filter paper and pooled after morphological classification depending on the species and life cycle stage. Collected adult ticks were identified as *H*. *longicornis*, *H*. *flava*, and *I*. *nipponensis*, and they were stored as one tick per tube. Collected nymphs were identified as *H*. *flava* and *H*. *longicornis* and were pooled from between 1 to 32 ticks per tube, by species. The only collected larvae species was *H*. *longicornis*, and 24 larvae were pooled in one tube. A total of 91 individual tick tubes and 28 pooled tick tubes were prepared. Genomic DNA was extracted from these samples, followed by N-PCR, targeting *Borrelia*-specific genes.

Classified ticks were added to a hard tissue grinding, MK28 tube (Bertin Technology, Rockville, MD, USA) containing 600 μL phosphate-buffered saline, with 10% fetal bovine serum and 5% penicillin/streptomycin, and ground using a FastPrep-24 Classic instrument (MP Biomedicals, Solon, OH, USA). Genomic DNA from the ground ticks was extracted with a G-spin Total DNA Extraction Kit (iNTRON Biotechnology, Sungnam-si, Korea) according to the manufacturer’s instructions.

N-PCR, targeting *Borrelia*-specific *pyrG* and *ospA* genes, was performed using genomic DNA from the tick samples, to detect *Borrelia* species. For the N-PCR targeting *pyrG*, *pyrG*-1F/*pyrG*-1R primers (primers for first PCR) and *pyrG*-2F/*pyrG*-2R primers (primers for N-PCR) were used [16]. For the N-PCR targeting *ospA*, Borrel-*ospA*F1/Borrel-*ospA*R1 primers (primers for first PCR) and Borrel-*ospA*F2/Borrel-*ospA*R2 primers (primers for N-PCR) were used [3]. To verify the PCR result, we conducted additional *flaB* N-PCRs, using primers Bmfla-1^st^-F/R (for the first PCR) and Bmfla-2^nd^-F/R (for the N-PCR) for *B*. *miyamotoi* identification, and primers BOR-fla-1F/1R (for the first PCR) and BOR-fla-2F/2R (for the N-PCR) for *Borrelia* spp. identification. All primer sequences and PCR cycling conditions are shown in Table 1. The PCR with AmpliTaq Gold 360 Master Mix (Applied Biosystems, Foster City, CA, USA) was performed in a Veriti 96-Well Thermal Cycler (Applied Biosystems). The 20 μL reaction mixture for the first PCR was composed of 1 μL each of 5 μM forward and reverse primers, 10 μL of Master Mix, 2 μL GC enhancer, 2 μL genomic DNA, and 4 μL distilled water. For N-PCR, the same reaction mixture as used for first PCR was used, except that the first PCR product was used as template DNA and the N-PCR primers were included. The PCR was performed using gene-specific PCR primers and annealing temperature, under the following conditions: 10 min at 94°C for pre-denaturation, 30 cycles of 20 s at 94°C, 30 s at each annealing temperature, 30 s ~ 1 min at 72°C and a final extension step of 7 min at 72°C. In each PCR run, a negative control (reaction mixture without the template DNA) was included. Genomic DNA of *B*. *burgdorferi* B31 Clone 5A1served as a positive control. PCR primers, annealing temperatures, and the amplicon sizes are shown in Table 1. The PCR products were examined by electrophoresis in a 1.2% agarose gel containing ethidium bromide.

**Table 1:** Oligonucleotide primers and PCR conditions used in this study.

Target gene^a^	Primer name (sequence)	Annealing temperature (°C)	Product size (bp)	References
*pyrG* nested PCR (external primer)	pyrG-1F (5’-ATTGCAA GTTCTGAGAATA-3’) pyrG-1R (5’-CAAACATTACGAGCAAATTC-3’)	45	801	[16]
*pyrG* nested PCR (internal primer)	pyrG-2F (5’GATATGGAAAATATTTTATTTATTG-3’) pyrG-2R (5’—AAACCAAGACAAATTCCAAG-3’)	49	707	[16]
*ospA* nested PCR (external primer)	Borrel-ospAF1 (5’-GGGAATAGGTCTAATATTAGC-3’) Borrel-ospAR1 (5’-CTGTGTATTCAAGTCTGGTTCC-3’)	52	427	[3]
*ospA* nested PCR (internal primer)	Borrel-ospAF2 (5’-CAAAATGTTAGTAGCCTTGAT-3’) Borrel-ospAR2 (5’-TCTGTTGATGACTTGTCTTT-3’)	52	314	[3]
*B*. *miyamotoi* *flaB* nested PCR (external primer)	Bmfla-1st-F (5’-AATCATAATACGTCAGCCATAAATG-3’) Bmfla-1st-R (5’- CATATTGAGGCACTTGATTTGC-3’)	55	976	This study
*B*. *miyamotoi* *flaB* nested PCR (internal primer)	Bmfla-2nd-F (5’-GTGGGTATAGAATTAATCGTGC-3’) Bmfla-2nd-R (5’- AAGATTTGCTCTTTGATCAGTTAC-3’)	56	701	This study
*Borrelia* spp. *flaB* nested PCR (external primer)	BOR-fla-1F (5’-ATACATCAGCTATTAATGCTTCAAG-3’) BOR-fla-1R (5’- GCTACAACCTCATCTGTCATTG-3’)	56	901	This study
*Borrelia* spp. *flaB* nested PCR (internal primer)	BOR-fla-2F (5’-GTGGTTAYAGAATTAATMGAGC-3’) BOR-fla-2R (5’-TAAATTTGCTCTTTGATCACTTATC-3’)	55	707	This study
16S rRNA conventional PCR	16S+1-F (5’-CTGCTCATGAATATTTAAATTGC-3’) 16S-1-R (5’-CGGTCTAAACTCAGATCATGTAGG-3’)	55	453	[17]

^a^*pyrG*, CTP synthase gene; *ospA*, outer surface protein A gene; *flaB*, flagellin gene; 16S rRNA, 16S ribosomal RNA gene

To identify tick species, conventional PCR (C-PCR), targeting mitochondrial 16S rRNA genes, was performed using the genomic DNA of ticks in which *Borrelia* spp. was detected [17]. C-PCR was performed with 16s+1-F/16S-1-R primers, AmpliTaq Gold 360 Master Mix (Applied Biosystems, CA, USA), and a Veriti 96-Well Thermal Cycler (Applied Biosystems, CA, USA), under the same condition as above. Primer sequences and PCR cycling conditions are presented in Table 1.

### Nucleotide sequencing

For DNA sequencing, the PCR products were purified using a QIAquick Gel Extraction Kit (QIAGEN, Hilden, Germany). The purified DNA fragments were then sequenced in both directions, using the PCR primers and an automatic sequencer (ABI Prism 3730XL DNA analyzer, Applied Biosystems) at Solgent (Deajeon, Korea). To identify the bacteria present, the sequencing results were analyzed using the BlastN program from the National Center for Biotechnology Information (NIH, Bethesda, MD, USA) website.

### Phylogenetic analysis

DNA sequences were identified and analyzed for homology comparisons using DNASTAR-Lasergene v6 software (DNASTAR, Madison, WI, USA) and the NCBI BlastN network service. The concatenating sequences of *pyrG* and *flaB* were aligned using Clustal W. A phylogenetic tree was constructed based on the DNA sequences of *pyrG* and *flaB* from tick samples with a *Borrelia* infection, and from various *Borrelia* strains in GenBank, using the neighbor joining (N-J) method on Clustal X and the Tree Explorer program (DNASTAR, Madison, WI). Bootstrap analysis was conducted using 1000 replicates to improve the confidence level of the phylogenetic tree. All sequence data generated in this study have been submitted to the NCBI GenBank (accession numbers MF948168 to MF948177 and MH102390 to MH102393).

## Results

A total of 484 ticks were collected, including 417 *H*. *longicornis*, 42 *H*. *flava*, and 25 *I*. *nipponensis*, which were identified by morphological analysis of life cycle stage and species, 88 of which were adults, 372 nymphs, and 24 larvae. Ticks were pooled after morphological classification by species and stage, genomic DNA extracted, and N-PCR performed, targeting the *Borrelia*-specific *pyrG* gene. Five *I*. *nipponensis* adults were PCR-positive, and DNA sequence analysis revealed the presence of *B*. *miyamotoi* in Chosun T5-30 and *B*. *afzelii* in Chosun T32, 35, 37, and 41 ticks. Of the total 484 collected ticks, the *Borrelia* infection rate, confirmed by *pyrG* N-PCR, was 1.03% (5/484), while that of only the 25 *I*. *nipponensis* was 20.0% (5/25). In addition, the infection rates of *B*. *miyamotoi* and *B*. *afzelii* in the 484 ticks were 0.21% (1/484) and 0.83% (4/484), respectively. To confirm these identification results with another target gene, we performed another N-PCR assay using genomic DNA from the five *Borrelia*-positive *Ixodes* ticks, targeting the *Borrelia*-specific gene *ospA*. Only 3 out of the 5 ticks, Chosun T32, 37, and 41, were PCR-positive, and DNA sequence analysis confirmed the presence of *B*. *afzelii*. To verify the results, we conducted *flaB* N-PCRs using the same *Borrelia*-positive tick genomic DNAs. The *B*. *miyamotoi*-specific *flaB* N-PCR, and sequencing analysis, confirmed the presence of *B*. *miyamotoi* in the Chosun T5-30 ticks. The *Borrelia* spp.-specific *flaB* N-PCR and sequencing analysis confirmed the presence of *B*. *afzelii* in only three ticks, Chosun T32, 37, and 41. Of the total 484 ticks collected, the infection rate of *Borrelia* species, with different confirmatory PCR testing, was 0.83% (4/484), while that of only the adult ticks was 4.55% (4/88), and more specifically, that of the *I*. *nipponensis* adult ticks was 16.0% (4/25). In addition, the confirmed infection rates of *B*. *miyamotoi* and *B*. *afzelii* in the 484 ticks were 0.21% (1/484) and 0.62% (3/484), respectively. The confirmed infection rates of *B*. *miyamotoi* and *B*. *afzelii* in only the adult ticks were 1.14% (1/88) and 3.41% (3/88), respectively, and of the 25 *I*. *nipponensis* collected, the infection rates of confirmed cases of *B*. *miyamotoi* and *B*. *afzelii* were 4.0% (1/25) and 12.0% (3/25), respectively (Table 2).

**Table 2:** *Borrelia* species identified by nested PCR for *pyrG*, *ospA*, and *flaB* in collected tick species.

Tick species	Ticks examined		Ticks with *B*. *miyamotoi*				Ticks with *B*. *afzelii* (%)			
	Stage	No.	*pyrG* N-PCR	*ospA* N-PCR	*flaB* N-PCR	Confirmed No. (%)	*pyrG* N-PCR	*ospA* N-PCR	*flaB* N-PCR	Confirmed No. (%)
*Haemaphysalis longicornis*	Adult	31	0			0	0			0
	Nymph	362	0			0	0			0
	Larva	24	0			0	0			0
*Haemaphysalis flava*	Adult	32	0			0	0			0
	Nymph	10	0			0	0			0
*Ixodes* *nipponensis*	Adult	25	1	0	1	1 (4)	4	3	3	3 (12)
**Total**	Adult	88	1		1	1 (1.14)	4	3	3	3 (3.41)
	Total	484	1		1	1 (0.21)	4	3	3	3 (0.62)

**Fig 1:**
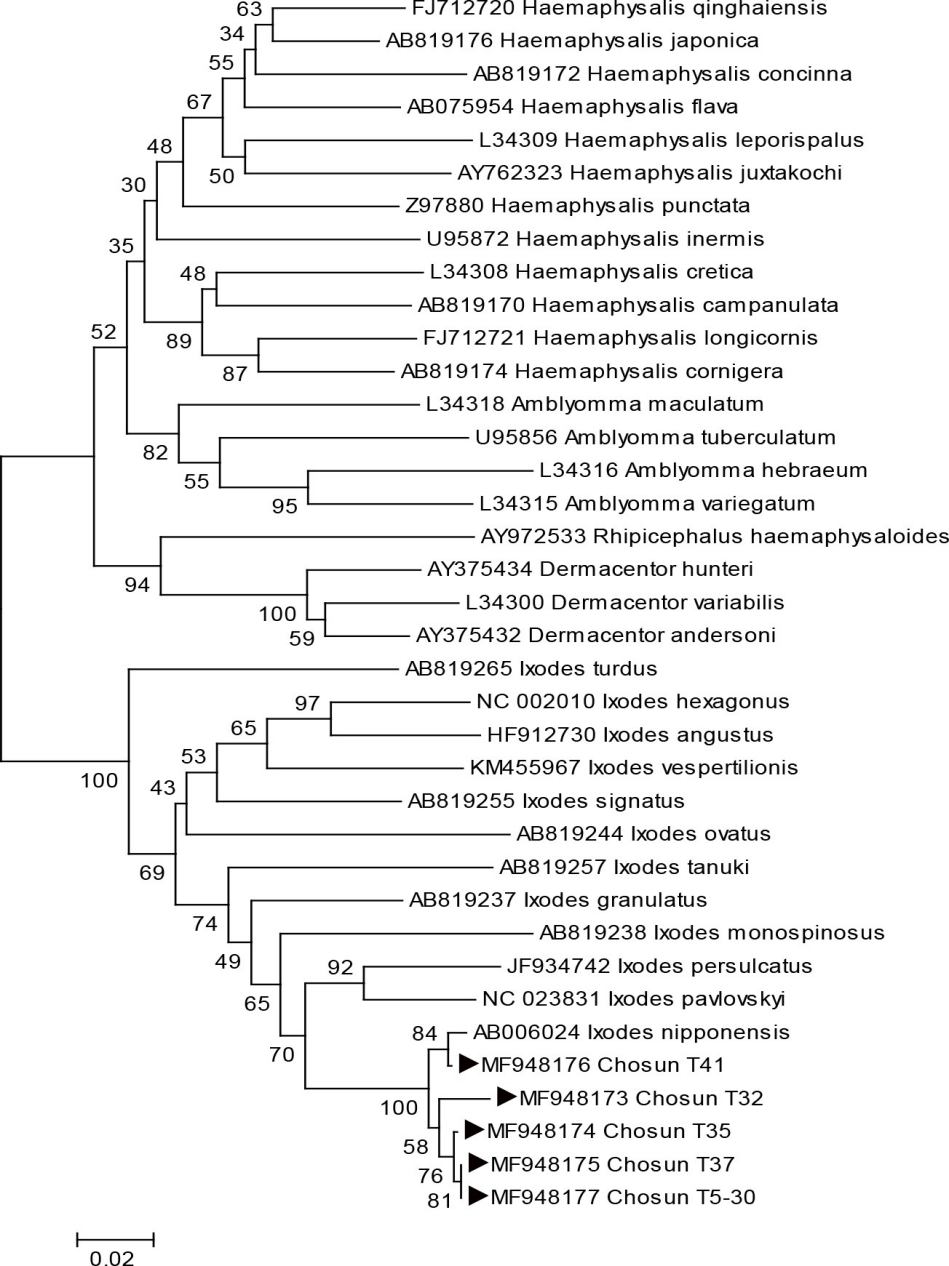
Phylogenetic tree based on 16S rRNA partial sequences (354 bp) from GenBank and *Borrelia*-positive tick specimens collected in this study (▶). Scale bars indicate 0.02 base substitutions per site. GenBank accession numbers are shown in the tree.

For the accurate genetic identification of ticks, conventional PCR (C-PCR), targeting 16S rRNA, was performed using genomic DNA from the five *Ixodes* ticks with *Borrelia*-positive results, then further investigated by DNA sequencing analyses. Molecular detection confirmed that all five ticks were *I*. *nipponensis*. A phylogenetic tree was constructed using DNA sequences of 16S rRNA gene fragments from five *Borrelia*-positive *I*. *nipponensis* tick samples (Chosun T32, 35, 37, 41, and 5–30) and using the 16S rRNA gene sequences of various ticks (*I*. *nipponensis*, *I*. *persulcatus*, *I*. *pavlovskyi*, *H*. *flava*, *H*. *longicornis*, *A*. *maculatum*) from GenBank as reference sequences. All sequences of Chosun T32, 35, 37, 41, and 5–30 ticks formed a cluster with *I*. *nipponensis* (Fig 1).

Homology testing showed that the 16S rRNA sequences of Chosun T5-30 and T32 ticks had a 99.8% and 98.4% homology with the 16S rRNA partial sequence of *I*. *nipponensis* mitochondrial DNA from adult males isolated in Japan, respectively, while all sequences from Chosun T35, 37, and 41 ticks showed a 99.1% homology.

**Fig 2:**
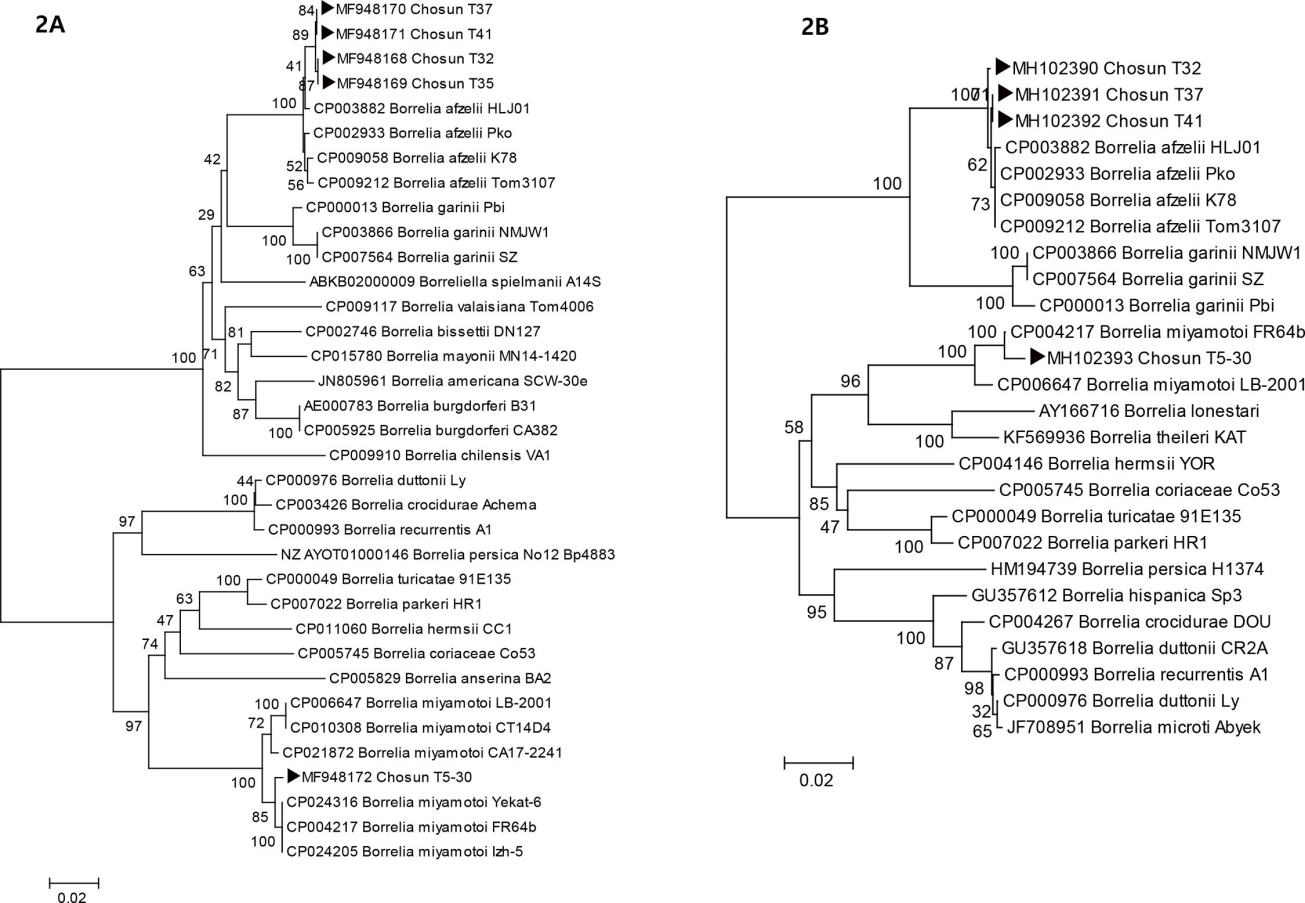
Phylogenetic tree based on partial *pyrG* sequences (A, 675 bp) and *flaB* sequences (B, 660 bp) from GenBank and *Borrelia*-positive tick specimens collected in this study (▶). Scale bars indicate 0.02 base substitutions per site. GenBank accession numbers are shown in the tree.

Another phylogenetic tree was constructed by concatenating DNA sequences of the *pyrG* (675 bp) and *flaB* (660 bp) genes from *Borrelia*-positive *I*. *nipponensis* tick samples, using various *Borrelia* strains (*B*. *burgdorferi* B31, *B*. *garinii* Pbi, *B*. *miyamotoi* FR64b, *B*. *afzelii* HLJ01) from GenBank as reference sequences. The phylogenetic trees generated from the *pyrG* and *flaB* gene sequences showed similar topologies with the sequences from Chosun T32, 35, 37, and 41 forming a cluster with *B*. *afzelii*, and the sequences from Chosun T5-30 forming a cluster with *B*. *miyamotoi* (Fig 2).

A homology search showed that *B*. *miyamotoi*, from Chosun T5-30, had a 99.4% sequence similarity with *B*. *miyamotoi* FR64b, which was isolated from the blood of *Apodemus argenteus* captured in Japan. *B*. *afzelii*, from the Chosun T32, 35, 37, and 41 ticks, showed 99.3%, 99.3%, 99.4%, and 99.4% sequence similarities with a *B*. *afzelii* HLJ01 isolate from Chinese patients, respectively.

## Discussion

After *B*. *miyamotoi* was first identified in *I*. *persulcatus* ticks and *Apodemus* mice, collected in Hokkaido, Japan [18], it was also found in several other tick species, such as *I*. *scapularis* and *I*. *pacificus* in the US, *I*. *ricinus* in Europe, and *I*. *persulcatus* in Europe and Asia [12, 18–20]. Crowder et al. reported the prevalence of *B*. *miyamotoi* in *Ixodes* ticks collected by flagging in the United States, Germany and the Czech Republic in Europe from 2008 through 2012. The infection of *B*. *miyamotoi* in ticks was found in 16 of the 26 sites surveyed and the incidence rate varied greatly by region; 1.8% for *I*. *ricinus* in Germany, 2% for *I*. *ricinus* in Czech Republic, 0~12.3% for *I*. *scapularis* and 0~15.4% for *I*. *pacificus ticks* in the United States, respectively [19]. Platonov et al. reported that *I*. *persulcatus* functions as a vector for *B*. *miyamotoi* in Russia [21]. According to a report on ticks from Hokkaido, Japan, which is geographically close to South Korea, overall tick infection rates of *B*. *miyamotoi*, the relapsing fever spirochete, were approximately 2% (71/3532) for *I*. *persulcatus*, 4.3% (5/117) for *I*. *pavlovskyi*, and 0.1% (1/676) for *I*. *ovatus* [22]. In addition to ticks, a retrospective surveillance of Lyme borreliosis in Japan detected *B*. *miyamotoi* DNA in the serum of two patients [23]. Moreover, emerging cases of relapsing fever, caused by *B*. *miyamotoi*, were reported in Russia, North America, and Europe, and in particular, *B*. *miyamotoi*-related meningoencephalitis was reported in a US patient [24]. The four Korean isolates of *B*. *burgdorferi* sensu lato, were first identified in *I*. *persulcatus* ticks and *Apodemus agrarius* rodent [14]. According to a report on ticks collected from humans, of the 261 ticks identified, the most abundant tick was *H*. *longicornis* (81.2%), followed by *Amblyomma testudinarium* (6.5%) and *I*. *nipponensis* (5.7%) [25]. Because *I*. *nipponensis* is the most frequently collected tick from wild rodents in South Korea, it is possible that patients bitten by *I*. *nipponensis* ticks are at a higher risk of infection by *Borrelia* spp. [26]. *B*. *miyamotoi* has, however, never been identified in South Korea nor its vector been reported; thus, the present study was conducted to investigate *Borrelia* infection in ticks collected in grasses, in South Korea. Of the 484 ticks, 417 (86.2%) were *H*. *longicornis*, 42 (8.7%) were *H*. *flava*, and 25 (5.2%) were *I*. *nipponensis*. All ticks that were positive for harboring *Borrelia* spp. were *I*. *nipponensis* adults, with infection rates of 4.0% for *B*. *miyamotoi* and 12.0% for *B*. *afzelii* being detected. The most common *Borrelia* spp. detected was *B*. *afzelii*, while *B*. *miyamotoi* was identified in South Korea. Prior to the identification of *B*. *miyamotoi*, only *B*. *burgdorferi* B31 Clone 5A1 was housed in our laboratory, which we included as a positive control, negating the chance of PCR contamination. Because *B*. *miyamotoi* has now been detected in South Korea, its reservoirs, rodents and other natural reservoirs, require further study. Because infection by *B*. *miyamotoi* results in non-specific fever symptoms, it is difficult to clinically differentiate this infection from anaplasmosis, Lyme diseases, and babesiosis. These three infectious diseases have been confirmed to occur in South Korea [27], and the present study confirmed the presence of *B*. *miyamotoi* in ixodid ticks. The detection of a pathogen in an individual, blood-feeding arthropod by no means proves or even suggests its role as a vector and a series of specific requirements would need to be examined to prove vector suitability. Although no patients have been reported to be infected by *B*. *miyamotoi* specifically, in South Korea, these results suggest that additional clinical studies should be undertaken to detect human infection cases of *B*. *miyamotoi*.

In conclusion, the present study detected *B*. *afzelii* and *B*. *miyamotoi* in *I*. *nipponensis* ticks, collected using a flagging and dragging method, in South Korea. Although only one *I*. *nipponensis* tick was positive for *B*. *miyamotoi*, our result suggests that *I*. *nipponensis*, confirmed by morphological and molecular biological methods, could potentially transmit *B*. *miyamotoi* to people in South Korea. Further collections in other areas of South Korea are necessary to establish whether *I*. *nipponensis* could viably serve as a vector of *B*. *miyamotoi* infections.
